# A Case of Status Epilepticus in a Patient Experiencing an Acute Attack of Hereditary Angioedema

**DOI:** 10.5811/cpcem.4816

**Published:** 2025-01-12

**Authors:** Danielle Weinberg, Steven Gayda, Kyle Hultz, Hanan Atia, Brian Kohen, Eric Boccio

**Affiliations:** * Memorial Healthcare System, Department of Emergency Medicine, Hollywood, Florida; † Memorial Healthcare System, Department of Pharmacy, Hollywood, Florida

**Keywords:** Hereditary angioedema, hereditary angioedema with normal C1-esterase inhibitor, HAE-nl-C1-INH, C1-esterase inhibitor, C1-INH, status epilepticus, case report

## Abstract

**Introduction:**

Hereditary angioedema (HAE) is a genetic disorder associated with recurrent episodes of angioedema in the absence of urticaria and pruritus. Hereditary angioedema is inherited in an autosomal dominant pattern and results in a quantitative deficiency (HAE type I) or dysfunction (HAE type II) of the C1-esterase inhibitor (C1-INH) protein. A very rare third type of HAE which is associated with normal quantitative and functional levels of C1-INH (HAE-nl-C1-INH) has been described.

**Case Report:**

A 54-year-old female with past medical history significant for HAE-nl-C1-INH presented to the emergency department (ED) for an acute attack of HAE and seizures. The patient arrived postictal after experiencing a total of three witnessed seizures, each lasting approximately 30 seconds. After the initial seizure was witnessed in the ED, the patient received 4200 Units of recombinant C1-INH intravenously. The patient’s mental status did not return to baseline, and she experienced two additional seizures. She was given a dose of the kallikrein inhibitor, ecallantide, as well as standard dosing of lorazepam and levetiracetam. The patient returned to her baseline and had no subsequent seizures while in the ED. Inpatient work-up included continuous video electroencephalography monitoring and magnetic resonance imaging of the brain, both of which were normal. The remainder of the inpatient course was uncomplicated, and the patient was discharged home neurologically intact.

**Conclusion:**

We present a case of status epilepticus in a patient with HAE-nl-C1-INH. The focus of emergent medical management of status epilepticus includes airway protection, respiratory support, and administration of abortive and prophylactic antiepileptic drugs. The emergency medicine physician should also consider and treat possible underlying etiologies. The treatment of an acute attack of HAE should focus on replacing C1-INH and preventing the formation and limiting the action of bradykinin.

## INTRODUCTION

Hereditary angioedema (HAE) has an estimated prevalence of 1 per 60,000 individuals and occurs equally between sexes assigned at birth, with the exception of HAE with normal C1-esterase inhibitor (HAE-nl-C1-INH), which is more prevalent in females.^1^,^2^ Hereditary angioedema types I and II are autosomal dominant diseases in which decreased quantitative or dysfunctional C1-esterase inhibitor (C1-INH) activity results in loss of regulation of the complement system, contact activating system, and the fibrinolytic pathway.

This leads to increased kallikrein-mediated cleavage of high molecular weight kininogen to form bradykinin, leading to vasodilation and swelling. HAE-nl-C1-INH is related to a variety of genetic mutations which can affect factor XII, plasminogen, angiopoietin-1, or high and low molecular weight kininogens, and is difficult to distinguish from types I and II, clinically.^3^ While the pathophysiology of HAE-nl-C1-INH is not completely understood, in many cases acute exacerbations appear to be associated with increased levels of estrogen, oral contraceptive usage, and pregnancy. Bradykinin is believed to be the primary mediator responsible for the development of angioedema during acute attacks.^4^

Clinically, HAE is characterized by recurrent episodes of angioedema in the absence of urticaria and pruritus. Commonly affected organ systems include the skin, respiratory and circulatory systems, and the gastrointestinal tract. Hereditary angioedema may cause facial and laryngeal swelling with subsequent airway obstruction, respiratory failure, and asphyxiation. Less common are central nervous system (CNS) manifestations. While there exists a paucity of reports in the literature, CNS manifestations of HAE include headaches, weakness and paresthesia, dizziness, visual disturbances, and seizures.^5^–^10^ The pathophysiology of HAE and how it affects the CNS is not well understood, although resultant cerebral edema has been posited. We present the first reported case of a patient experiencing an acute attack of HAE-nl-C1-INH and subsequently experiencing status epilepticus. The authors hope that this case report will serve as a general review of HAE-nl-C1-INH, raise awareness of the possibility of CNS manifestations of HAE, and provide an overview of the prophylactic and on-demand pharmacologic management for HAE.

**CPC-EM Capsule**

What do we already know about this clinical entity?

*Albeit rare, hereditary angioedema (HAE) can affect the central nervous system (CNS) manifesting clinically as headache, weakness, paresthesia, encephalopathy, and seizure*.

What makes this presentation of disease reportable?

*We present the first case of a patient suffering from status epilepticus secondary to HAE with normal C1-esterase inhibitor and outline the recommended therapeutic management options*.

What is the major learning point?

*Therapeutic management of HAE with CNS involvement includes C1 esterase inhibitors, bradykinin-2 receptor antagonists, kallikrein inhibitors, and fresh frozen plasma*.

How might this improve emergency medicine practice?

*The initial treatment of HAE with CNS involvement including status epilepticus is more complex than that secondary to generalized seizure disorders*.

## CASE REPORT

A 54-year-old female with past medical history significant for HAE-nl-C1-INH receiving treatment with lanadelumab-flyo, a monoclonal antibody targeting kallikrein, 300 milligrams (mg) subcutaneously (SQ) every two weeks for prophylaxis presented to the emergency department (ED) by ambulance for an acute attack of HAE and seizures. The family reported to emergency medical services (EMS) that the patient was suffering from edema of the face and upper neck and had begun experiencing full body tremors, tongue biting, and extensor posturing. Review of the patient’s electronic health record was significant for previous ED presentations for HAE attacks marked by facial and neck angioedema, syncope, visual disturbances, and generalized tonic-clonic seizures complicated by status epilepticus refractory to antiepileptic drugs and benzodiazepines requiring intubation and admission to the medical intensive care unit. Inpatient and outpatient neurology workups for underlying generalized seizure disorder were all unremarkable. Initial vital signs were remarkable for a blood pressure of 152/82 millimeters of mercury, heart rate of 130 beats per minute, and respiratory rate of 26 breaths per minute. The patient’s peripheral oxygen saturation was 100% while wearing a non-rebreather mask with oxygen flow rate at 15 liters/minute, and the patient was afebrile. The airway was protected and free of secretions. The face and upper neck were swollen while the lips, tongue, uvula, and posterior oropharynx were normal in appearance. Breath sounds were clear to auscultation in all lung fields, and there was no stridor. There was no evidence of tongue laceration, urinary incontinence, or trauma, and the skin was free of urticaria. The patient’s past medical history was corroborated by the family at bedside who also presented a letter from the patient’s immunologist describing the typical presentation of symptoms and outlining the recommended therapeutic management of HAE-nl-C1-INH attacks with neurologic involvement. According to these recommendations, in the event of cognitive or motor function decline, loss of consciousness, or laryngeal edema, ecallantide 30 mg SQ should be prioritized as first line treatment. Should symptoms continue 30 minutes after receiving ecallantide, recombinant C1-INH (rC1-INH) 4200 units (U) intravenous (IV) or icatibant 30 mg SQ was recommended. A repeat dose of each medication could be given 40–60 minutes following the initial dose, not to exceed more than two doses within 24 hours.

Emergency medical services administered the patient’s home on-demand abortive medications for HAE which included ecallantide 30 mg SQ and icatibant 30 mg SQ en route. After arrival, the patient had a seizure lasting approximately 30 seconds followed by a postictal period characterized by psychomotor retardation, confusion, and inappropriate word use without a return to baseline mental status. After the initial seizure, rC1-INH 4200 U IV was administered. The patient’s mental status did not return to baseline, and she experienced two additional seizures each lasting approximately 30 seconds. She was given a second dose of ecallantide in addition to what was given by EMS as well as lorazepam 8 mg IV and levetiracetam two grams IV. All seizure activity resolved approximately 10 minutes after administration of the second dose of ecallantide. The patient returned to her baseline and had no subsequent seizures while in the ED. Initial diagnostic testing included a basic metabolic panel and complete blood count which were within normal limits. C1-INH level and factor XII assay were normal. Non-contrast computed tomography (CT) of the brain performed in the ED demonstrated no acute processes. Inpatient work-up included continuous video electroencephalography (EEG) monitoring and a non-contrast magnetic resonance imaging (MRI) of the brain, both of which showed no acute abnormalities. The remainder of the inpatient course was uncomplicated. The patient’s mental status remained at baseline with no recurrence of seizures, the upper neck edema resolved while the facial edema improved, and the patient was discharged home neurologically intact.

## DISCUSSION

Central nervous system involvement in HAE is not well understood nor easily diagnosed. In the absence of advanced neuroimaging, central nervous system symptoms of HAE attacks may mimic those of a generalized seizure disorder. Diagnosis is often clinical and relies on understanding the history, risk factors, and resolution of symptoms following administration of therapeutic medications aimed at treating HAE.^11^,^12^ Neuroimaging such as CT brain and MRI brain are not sensitive for HAE.^7^ Cerebral edema manifests as hypodensities and loss of differentiation between gray and white matter on CT brain and appears as a bright signal on T2-weighted imaging and FLAIR pulse series and a low signal on T1-weighted imaging on MRI brain.

According to the United States Hereditary Angioedema Association Medical Advisory Board 2020 Guidelines for the Management of Hereditary Angioedema, medications approved for HAE focus on long and short-term prophylaxis and on-demand treatment for acute attacks.^13^ United States Food and Drug Administration (FDA)-approved medications for HAE prophylaxis include plasma-derived nanofiltered C1-INH (pdC1-INH) and plasma kallikrein inhibitors such as lanadelumab-flyo. Attenuated androgens such as danazol and stanozolol are no longer considered first line agents, and antifibrinolytics such as epsilon aminocaproic acid are no longer approved for HAE treatment.^14^ For on-demand treatment of HAE attacks, ecallantide, icatibant, pdC1-INH, and rC1-INH can be administered. Whereas icatibant, pdC1-INH, and rC1-INH can be self-administered, ecallantide cannot.^13^ Fresh frozen plasma (FFP) contains C1-INH and may be given if none of the FDA-approved on-demand medications are available, however, it should be noted that FFP also contains factor XII, prekallikrein, and high-molecular-weight kininogen which may lead to the increased production of bradykinin thus worsening symptoms of HAE.^15^,^16^ The For Angioedema Subcutaneous Treatment (FAST) 2 trial comparing icatibant to tranexamic acid (TXA) in patients with HAE presenting with cutaneous or abdominal attacks revealed a median time to first improvement of symptoms as assessed by patients and investigators of 12.0 hours for the TXA group suggesting that is has no role in the treatment of acute attacks.^17^ Additionally, patients on TXA for long-term prophylaxis did not experience a significant decrease in the number or duration of HAE attacks per year compared to controls.^18^

The use of multiple on-demand agents with varying mechanisms of action and at higher than recommended doses may increase the likelihood of aborting an HAE attack. The patient received ecallantide and icatibant during EMS transport and additional ecallantide and rC1-INH upon arrival to the ED. Ecallantide is relatively fast acting with an initial onset of 10–30 minutes and reaches a peak concentration at two to three hours.^19^ The CT and MRI brain imaging and EEG were all unremarkable, therefore cerebral edema secondary to an HAE exacerbation cannot be confirmed as the cause of the patient’s seizures; however, given the patient’s history of similar presentation, presence of facial and upper neck edema without urticaria or pruritis, and the observation that the seizures broke approximately 10 minutes following administration of the second dose of ecallantide, status epilepticus secondary to HAE-nl-C1-INH is likely. These observations support the use of fast acting kallikrein inhibitors as first line agents in the management of CNS manifestations of HAE-nl-C1-INH.

## CONCLUSION

Central nervous system symptoms of type I and II HAE have been reported in the allergy and immunology and neurology literature.^5^–^8^,^10^ To the best of our knowledge, this case is the first to describe status epilepticus during an HAE-nl-C1-INH attack. Given the diagnostic challenges of HAE-nl-C1-INH including the lack of provider awareness, clinical similarities shared with other types of angioedema, and limited availability of necessary diagnostic tests, its incidence is most likely underreported. Further research investigating the prevalence and pathophysiology of CNS manifestations of HAE across all subtypes is warranted.
